# Tuberculous mastitis, the great imitator in breast disease: a case report with diagnostic and therapeutic challenges

**DOI:** 10.1186/s40249-025-01391-9

**Published:** 2026-01-13

**Authors:** Serena Vita, Claudia Piccolo, Gaetano Maffongelli, Alberta Villanacci, Nicoletta Fusco, Laura Scorzolini, Claudia Palazzolo, Ada Petrone, Angela Corpolongo, Carla Nisii, Fabrizio Albarello, Beomonte Zobel, Francesca Svegliati, Franca Del Nonno, Daniele Colombo, Fabio Di Cesare, Emanuele Nicastri, Stefania Ianniello

**Affiliations:** 1 National Institute for Infectious Diseases ‘Lazzaro Spallanzani’ IRCCS, Via Portuense 292, 00149 Rome, Italy; 2 Department of Radiology, Fondazione Policlinico Campus Bio-Medico, via Alvaro del Portillo 21, 00128 Rome, Italy; 3 Oncology Unit, San Camillo-Forlanini Hospital, c.ne Gianicolense, 00152 Rome, Italy

**Keywords:** Breast, Tuberculous mastitis, Abscess

## Abstract

**Background:**

Extrapulmonary tuberculosis (TB) remains a diagnostic challenge due to its nonspecific clinical manifestations, low bacterial burden, and the need for invasive procedures to obtain diagnostic samples. Breast TB, a particularly rare form, can closely mimic other conditions such as inflammatory breast carcinoma.

**Case presentation:**

A 30-year-old Peruvian woman with no relevant medical history was admitted with painful erythema of the right breast and two fistulous tracts secreting purulent discharge. Initial microbiological tests, including interferon-gamma release assay (IGRA) and cultures, were negative. Imaging revealed multiple pulmonary micronodules, and breast biopsy demonstrated granulomatous inflammation with Langhans giant cells. Despite negative PCR and culture results for mycobacteria, empirical antitubercular therapy was initiated based on clinical, radiological, and histopathological evidence. The patient was initiated on standard antitubercular therapy, which was simplified two months later. Progressive clinical improvement was observed, with complete ulcer healing by month 4 and resolution of pulmonary nodules by month 7. By month 12, she remained in good clinical condition with no signs of recurrence.

**Conclusions:**

This case highlights the diagnostic complexity of breast TB and underscores the importance of a multidisciplinary approach in the absence of microbiological confirmation. Clinical context, imaging, and histology may guide successful empirical treatment and improve patient outcomes.

**Graphical Abstract:**

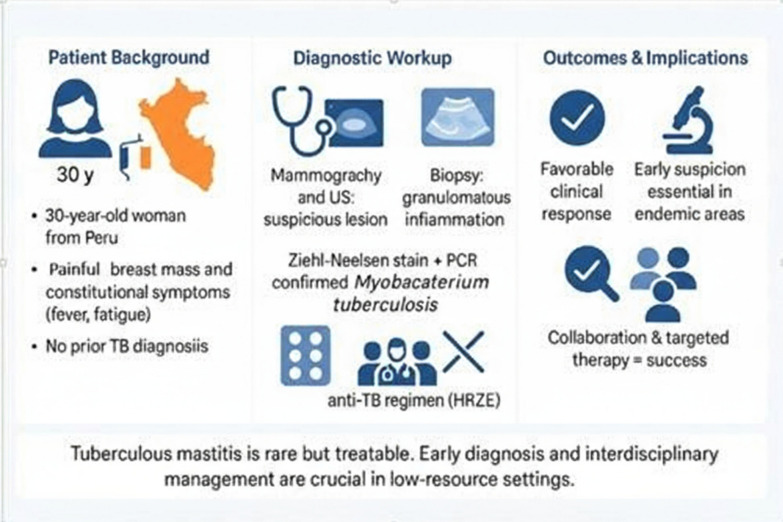

## Background

Tuberculosis (TB) is an infectious disease caused by *Mycobacterium tuberculosis*, primarily affecting the lungs. Its incidence is particularly high in developing countries, especially among immunocompromised individuals [1]. According to the most recent Global Tuberculosis Report (2024) published by the World Health Organization (WHO), TB likely re-emerged as the leading cause of mortality globally from a single infectious agent in 2023, following a three-year period during which COVID-19 temporarily assumed that position. TB was responsible for nearly twice the number of deaths as HIV/AIDS. Over 10 million individuals continue to develop TB annually, with incidence rates increasing since 2021 [2, 3]. The lungs represent the primary site of infection in tuberculosis. Among all extrapulmonary manifestations, tuberculous mastitis (TM) is a rare condition, with an estimated prevalence of approximately 0.1% [4]. Diagnosis requires a high degree of clinical suspicion due to the overlap of symptoms and radiological features with other breast lesions, including malignancies. Breast tissue provides resistance to the survival and multiplication of tuberculous bacilli and breast involvement is a rare clinical entity, even in endemic countries [5]. The nonspecific clinical and imaging features, combined with clinicians’ limited confidence in diagnosing this condition, contribute to high rates of misdiagnosis. It is often mistaken for breast cancer or a pyogenic breast abscess, complicating accurate diagnosis. [6]. McKeown et al. first described the five main types of infection within the breast: nodular mastitis, disseminated mastitis, sclerosing mastitis, mastitis obliterans, and acute miliary mastitis [7]. Thus, diagnosing TM presents a diagnostic challenge and may be delayed if patient risk factors and clinical history are not carefully considered. This case discusses the clinical presentation, diagnostic process, and therapeutic management, emphasizing the diagnostic challenges encountered in achieving an accurate diagnosis.

## Case presentation

### Patient demographics and medical history

A 30-year-old woman from Peru with no significant medical history was admitted to the Spallanzani Institute for Infectious Diseases in Rome in late November 2023 for assessment of a painful red lesion on her right breast.

### Symptoms and signs

On admission, the patient was stable but had painful redness of the right breast and two fistulas draining pus.

### Prior medical interventions

The patient was previously hospitalized elsewhere in November 2023, for the same condition. Interferon-gamma release assay (IGRA, QuantiFERON-Plus) and bacterial cultures were negative during that stay. She had diffuse erythema nodosum on all limbs and received intravenous amoxicillin-clavulanate (1 g three times daily) and daptomycin (750 mg once daily quam die) for 15 days.

### Recurrence and further evaluation

**Fig. 1:**
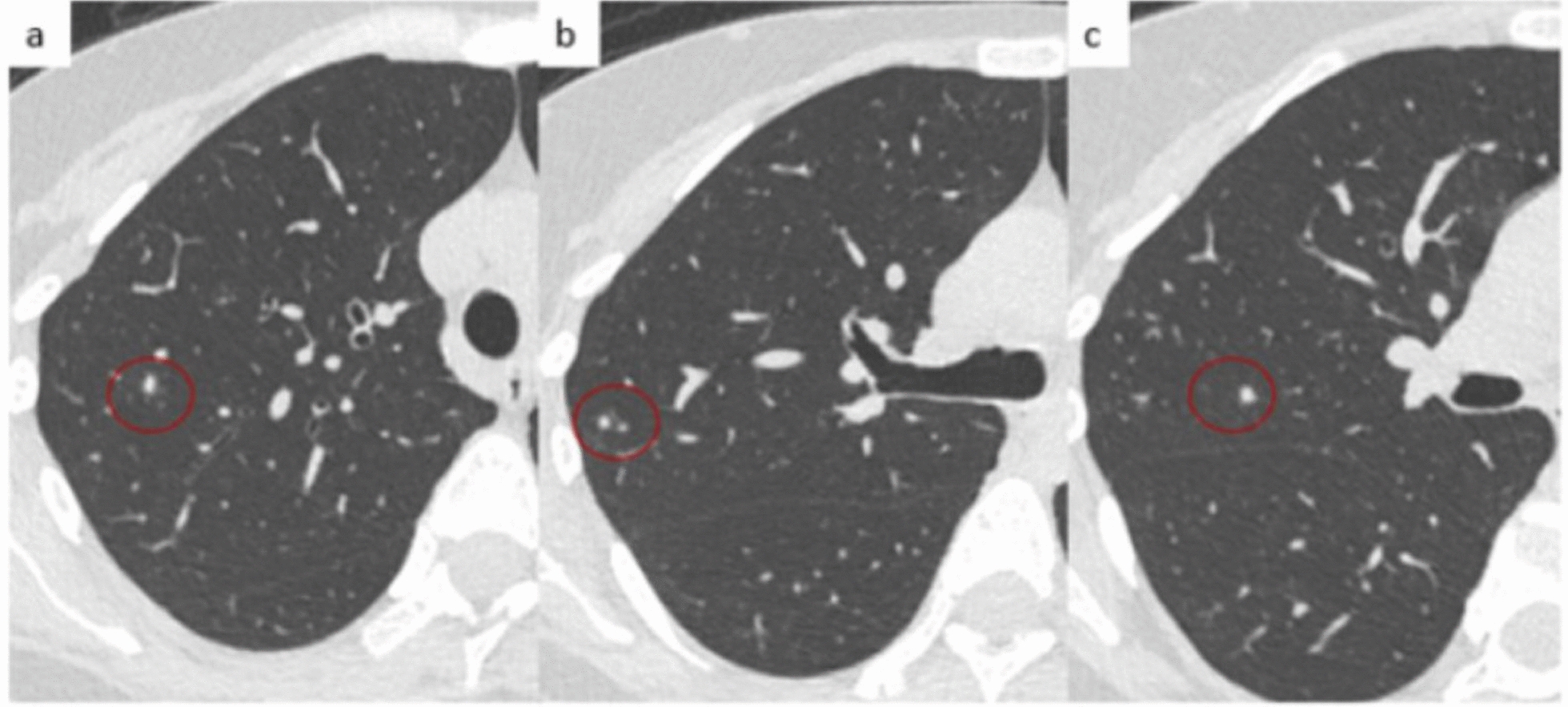
Computed tomography (CT) at diagnosis before starting therapy: the non-contrast CT scan shows the presence of solid, non-calcified nodules in the dorsal segment of the right upper lobe, without evidence of cavitation or surrounding ground-glass opacity

On November 28, 2023, 3 days post-discharge, she returned to the emergency department with recurring symptoms and was referred to the Spallanzani Institute. *Staphylococcus epidermidis* was repeatedly isolated from cultured secretion samples from fistulous tract but not from blood. A tuberculous infection was suspected after a chest computed tomography (CT) scan showed five micronodules in the apico-posterior segment of the right upper lobe, the largest being approximately 5 mm in diameter (Fig. 1) and at least six micronodules up to 4 mm in the left upper lobe.

### Microbiological and molecular assay

Respiratory secretion and breast samples from fistulous tract were processed using Ziehl-Neelsen staining, culture on Mycobacteria Growth Indicator Tube (MGIT) and Lowenstein-Jensen media (BD, Liofilchem, Italy), and real-time PCR with the Anyplex MTB/NTM Detection Kit (Seegene, Republic of Korea); all tests yielded negative results for *M. tuberculosis*.

### Therapeutic management

**Fig. 2:**
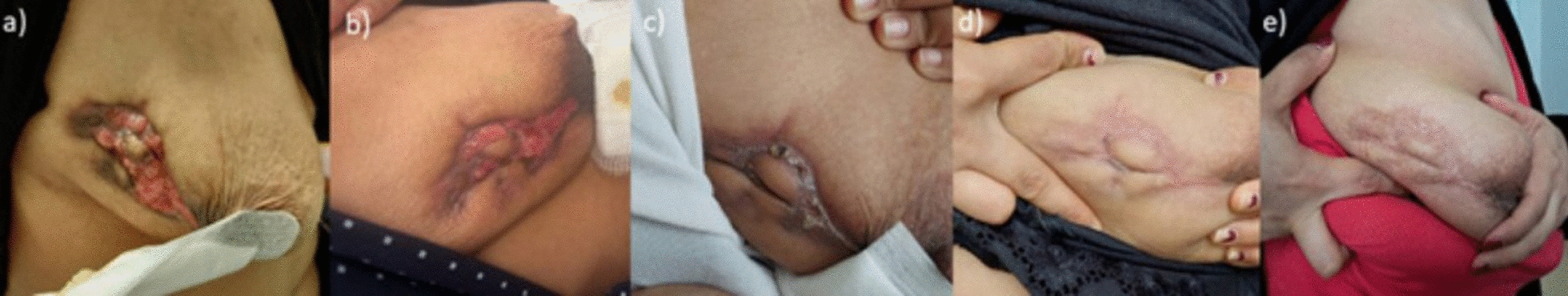
Breast lesion **a** before starting anti TB-therapy; **b** 20 days, **c** 2 months, and **d** 4 months and **e** 12 months after starting therapy

Empirical antibiotic therapy was initiated with intravenous daptomycin (750 mg once daily) and piperacillin/tazobactam (4.5 g three times daily) for 14 days. This treatment led to the resolution of discharge, although an ulcerative lesion persisted (Fig. 2a).

### Histopathological assessment

**Fig. 3:**
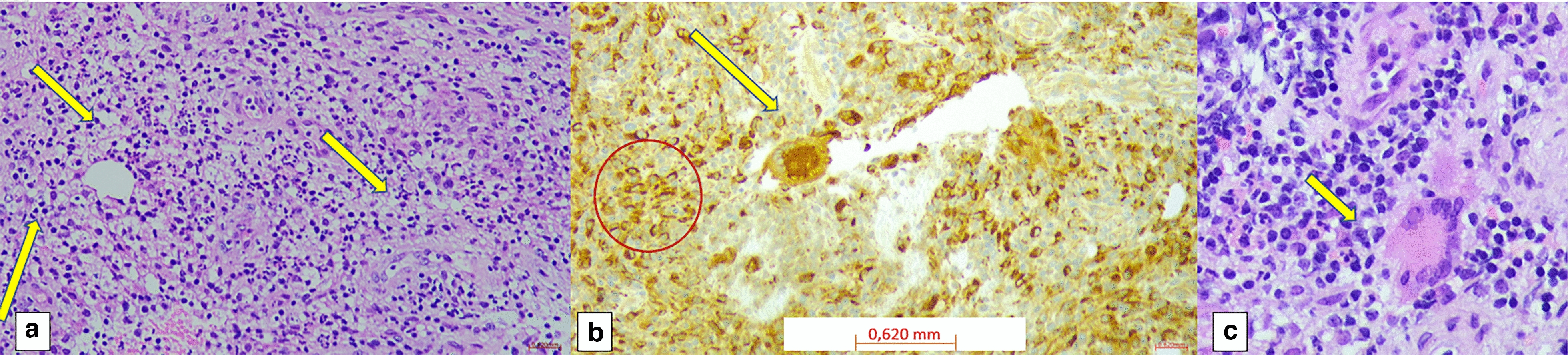
Fine needle aspiration of breast abscess: cytologic evaluation performed on cell block preparation; cytologic material was collected and processed as a paraffin embedded block (Agar based method) **a** Acute and chronic inflammation with several neutrophils (*yellow arrows*) and macrophages (hematoxylin and eosin stain, ×100 original magnification, OM). **b** Macrophages (*red circle*) and Langhans giant cell (*arrow*) highlighted by anti-CD68 antibody (anti-CD68 clone KP-1, ×100 OM). **c** Langhans giant cell (*arrow*) within acute and chronic inflammation (hematoxylin and eosin, ×400 OM). Scale bar 100 pixel = 100 µm

Cytology of the ulcer showed granulomatous inflammation with Langhans giant cells and epithelioid histiocytes. Cytology of the ulcerated area revealed granulomatous inflammation with Langhans giant cells and epithelioid histiocytes (Fig. 3). To further characterize the lesion, a vacuum-assisted breast biopsy (VABB) under ultrasound guidance was performed. This technique allows multiple, larger tissue samples with minimal invasiveness and high diagnostic accuracy. Histological examination showed chronic granulomatous inflammation with Langhans cells, neutrophils, eosinophils, lipid phagocytosis, and fibrosis. PCR and culture of biopsy samples were negative for mycobacteria, including broad-range 28S PCR analysis.

### Magnetic resonance imaging (MRI) protocol

**Fig. 4:**
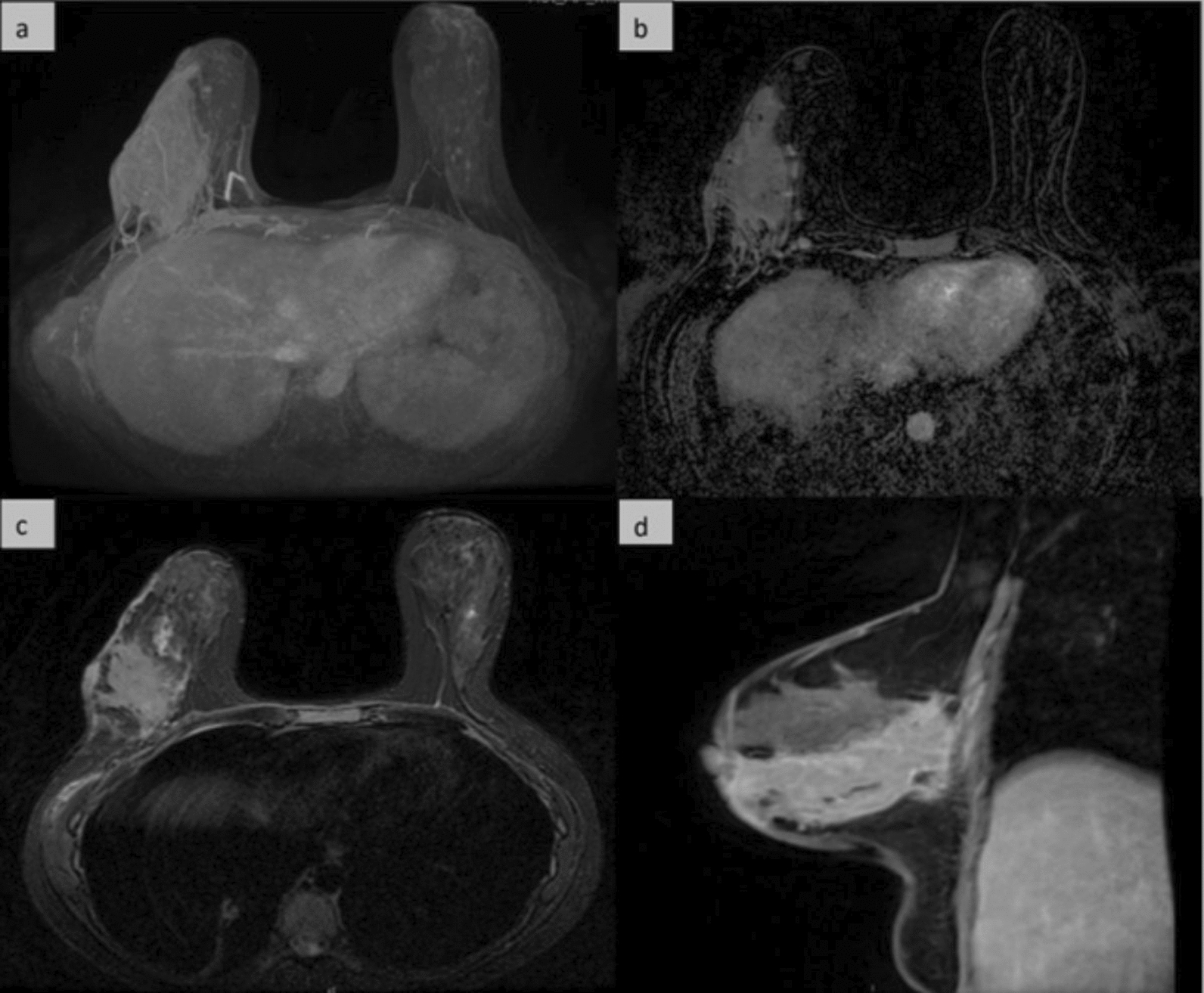
Magnetic resonance imaging at diagnosis before starting therapy: **a** MIP on axial plane, **b** post contrast subtracted T1-weighted image with fat saturation and **c** and no-subtracted on axial plane and **d** sagittal plane show an extensive soft tissue enhancing area involving the outer quadrants of the right breast, from the nipple to the deep planes posteriorly, with skin thickening. *MIP* Maximum intensity projection

On December 15, 2023, a contrast-enhanced breast MRI demonstrated skin thickening and a large (12 × 5.3 cm) lesion extending from the areola-nipple complex to deep muscle planes with diffuse hypervascularization (Fig. 4). MRI was performed with dedicated bilateral breast coils, using T1-weighted, T2-weighted (with/without fat suppression), diffusion-weighted (b = 0, 800–1000), and dynamic contrast-enhanced (DCE) sequences after gadobutrol (0.1 mmol/kg) injection. Post-processing included maximum intensity projection (MIP) and subtracted fat-suppressed T1 images, with slice thickness 1–3 mm and temporal resolution ≤ 90 s per post-contrast phase.

### Diagnosis and empirical antitubercular therapy

**Fig. 5:**
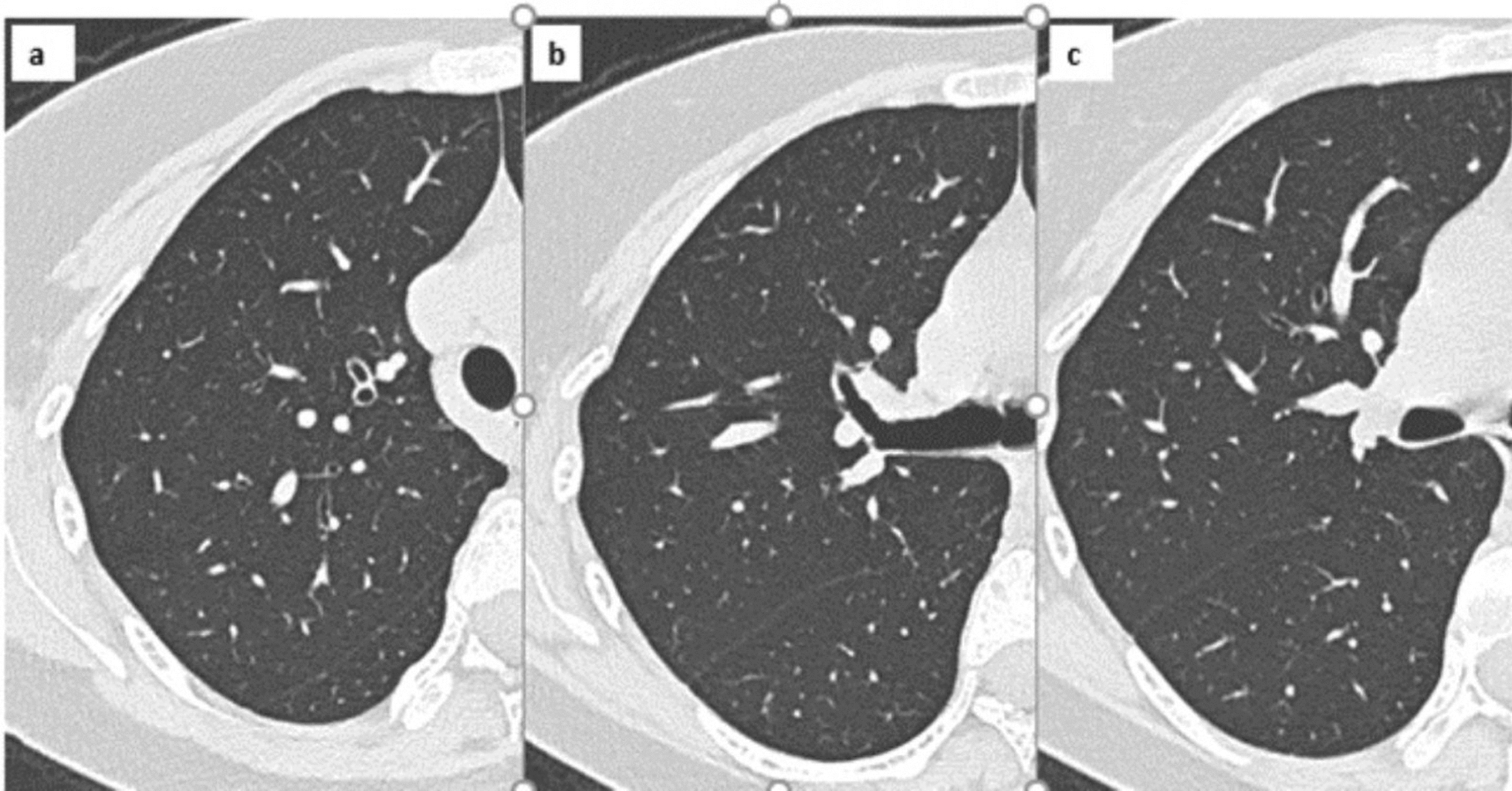
The non-contrast computed tomography scan performed after 7 months shows complete resolution of the pulmonary findings

Given the incidence rate of tuberculosis in the patient’s country of origin, the clinical failure of combined antibiotic therapy, as well as radiological and histopathological findings strongly indicative of tuberculous infection, empirical oral antitubercular treatment was initiated. The standard regimen included isoniazid 300 mg, rifampicin 600 mg, pyrazinamide 2000 mg, and ethambutol 1200 mg all orally administered daily. Two months later, it was simplified to isoniazid and rifampicin, only (Fig. 5).

### Outcome and follow-up

**Fig. 6:**
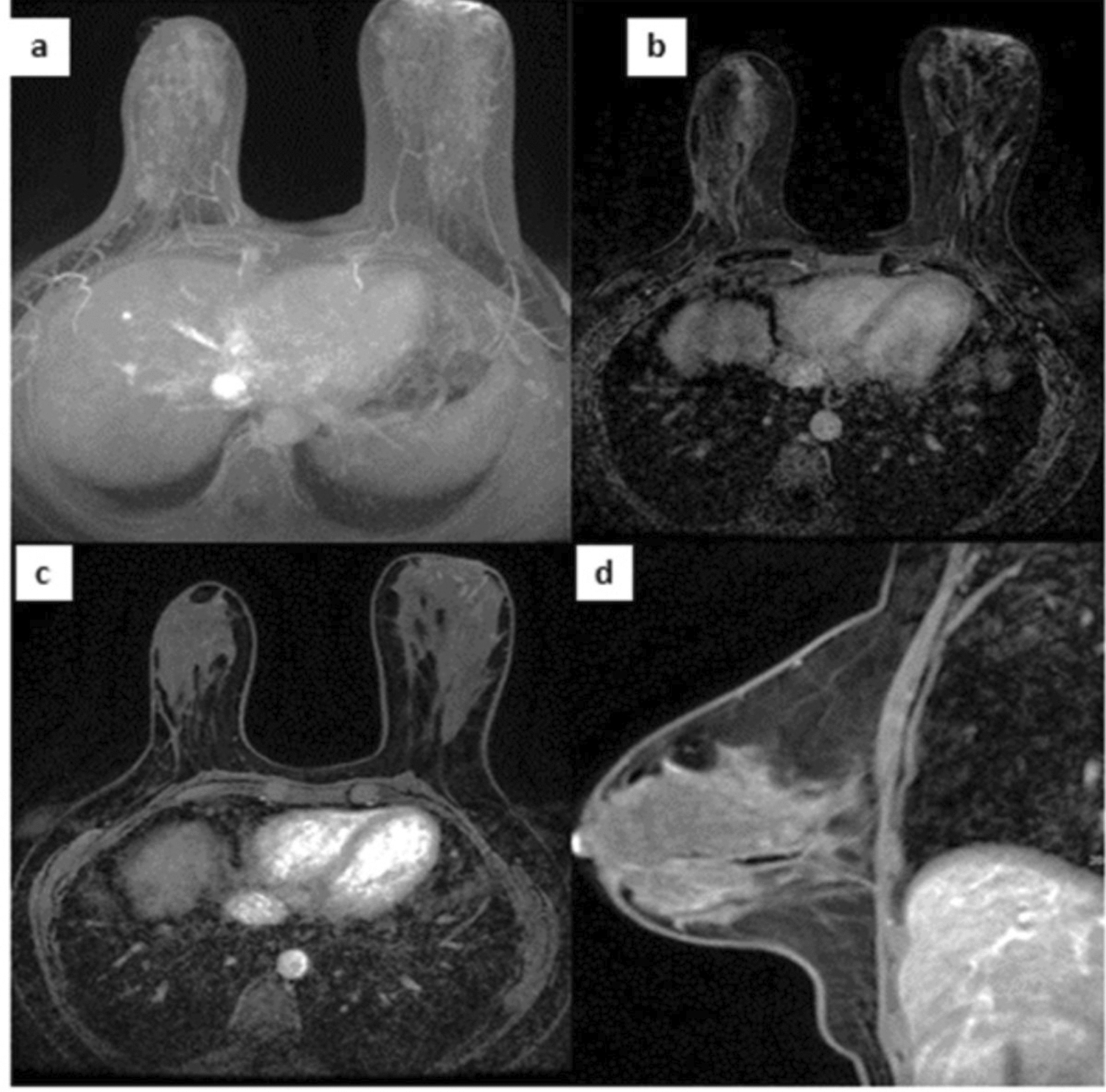
Magnetic resonance imaging performed 9 months after start of TB therapy: **a** MIP on axial plane, **b** post contrast subtracted T1-weighted image with fat saturation and **c** and no-subtracted on axial plane and **d** sagittal plan show a significative reduction in size of the enhancing tissue, with multiple and confluent foci of contrast enhancement. The skin thickenning has also disappeared. *MIP* Maximum intensity projection

One month after treatment initiation, ulcer healing was remarkable with complete re-epithelialization by month 4 (Fig. 2d). At month 7, follow-up chest CT confirmed resolution of pulmonary micronodules, and by month 9, breast MRI showed no abnormal enhancement—only residual fibrotic tissue with mild late enhancement (Fig. 6). At month 12, the patient remained asymptomatic with no recurrence or adverse effects (Fig. 2e).

## Discussion and conclusions

First described in 1829 by English surgeon Astley Cooper, TM is a rare manifestation of extrapulmonary *M. tuberculosis* infection, accounting for approximately 0.06–0.1% of all presentations [4]. It is referred as the ‘great impostor’ because it may lead to misdiagnosis as a chronic abscess, granulomatous mastitis, or breast carcinoma without a primary tubercular complex elsewhere in the body [8]. Literature data are limited, but its highest prevalence is primarily among young, multiparous, and lactating women likely due to duct dilation and increased blood supply, making them more susceptible to the infection. The breast infection can spread hematogenously and lymphatically, originating from axillary lymph nodes, duct lesions, or direct extension from contiguous structures. The infection is generally considered secondary, although the primary complex remains obscure; the few cases described as primary infections are due to breast infections through skin lesions on the nipple and/or major glandular ducts [9].

### Diagnostic challenge

This case highlights the diagnostic challenges posed by tuberculous mastitis, a rare yet significant clinical entity. The patient, a young Peruvian woman with a history of recurrent breast abscesses, presented with painful inflammatory breast disease that repeatedly relapsed despite multiple courses of antibiotics. The intrinsic resistance of breast tissue to tuberculous bacilli, along with nonspecific clinical presentations and imaging characteristics, underscores the importance of a high index of suspicion in endemic regions and among patients from high incidence areas. Multiple microbiological tests—including sputum cultures, breast secretion cultures, and PCRs—were negative, and IGRA was non-reactive. Such negative results could have discouraged further suspicion, but the combination of epidemiological context (origin from a TB-endemic country), radiological findings (pulmonary micronodules), histological features (granulomatous inflammation with Langhans giant cells), and failure of conventional antibiotics strongly supported tuberculosis as the underlying cause.

#### The role and limitations of IGRA

In our case, IGRA was negative at initial presentation. This result does not exclude active tuberculosis, since IGRAs have limited sensitivity in this context. Reported sensitivities for active disease range between 58 and 78% across studies, with even lower performance in extrapulmonary and pediatric cases [10, 11]. Current guidelines emphasize that IGRAs should not be used as standalone diagnostic tools to rule out active tuberculosis [12]. Therefore, a negative IGRA should never override a strong clinical suspicion, particularly in patients from endemic areas or with extrapulmonary manifestations.

#### Histology and imaging and empirical treatment

MRI can help determine the extent of the disease into the thoracic wall and possible involvement of bone, lymph nodes, or muscle structures, but the gold standard for diagnosis remains the culture of *Mycobacterium* with Ziehl-Neelsen staining [13]. PCR, fine-needle aspiration cytology, and breast biopsy can assist in the diagnostic process, but individually, do not provide a definitive diagnosis. Nevertheless, histopathological evidence of granulomatous inflammation with epithelioid histiocytes and Langhans giant cells represents a crucial clue that, in the appropriate clinical and epidemiological context, may strongly support the initiation of empirical antitubercular therapy.

McKeown described five distinct forms of tuberculous mastitis: nodular, disseminated, sclerosing, obliterans, and acute miliary. Our patient’s presentation, with diffuse erythema, painful induration, and fistulizing tracts extending into deep planes, is most consistent with the disseminated form. Recognizing these patterns is essential to avoid misdiagnosis, especially when microbiological confirmation is lacking.

Surgical treatment does not seem to offer better benefits than conservative approaches and is generally used for draining abscesses or removing masses in cases of poor therapy response [14]. Despite negative initial tests, the clinical improvement following antitubercular treatment supports the diagnosis of tuberculous mastitis. This reinforces the importance of integrating epidemiology, imaging, histology, and treatment response in guiding decision-making.

#### Take-home message

Tuberculous mastitis, though rare, poses a major diagnostic challenge due to its clinical and radiological similarities to breast cancer and other chronic infections. This case demonstrates that negative microbiological tests should not exclude tuberculosis when epidemiological, radiological (such as MRI and CT), and histopathological findings converge. A high index of suspicion, particularly in patients from endemic areas, and close multidisciplinary collaboration are essential to ensure timely diagnosis and treatment. Due to its uncommon and often atypical presentation, increasing clinician awareness is crucial to facilitate timely diagnosis and management, thereby minimizing the risk of complications.

## Data Availability

All data generated or analyzed relating to this study are presented within this published article.

## Abbreviations

TM: Tuberculous mastitis
IGRA: Interferon-gamma release assay
CT: Computed tomography
MRI: Magnetic resonance imaging
OM: Original magnification
